# Perspective-taking with affected others to promote climate change mitigation

**DOI:** 10.3389/fpsyg.2023.1225165

**Published:** 2023-09-28

**Authors:** Ann-Kathrin Koessler, Nicolai Heinz, Stefanie Engel

**Affiliations:** ^1^Institute of Environmental Planning, Leibniz University Hannover, Hannover, Germany; ^2^School of Business Administration and Economics and Institute for Environmental Systems Research (IUSF), Osnabrück University, Osnabrück, Germany; ^3^Environmental Politics, Helmholtz Centre for Environmental Research (UFZ), Leipzig, Germany

**Keywords:** perspective-taking, pro-environmental behavior, climate change, empathy, experiment

## Abstract

Prior evidence suggests that perspective-taking may promote pro-environmental behavior, at least for low-cost behaviors or local environmental problems. Climate change, however, requires costly mitigation efforts and is a global problem. Thus, in this study, we examine whether perspective-taking in the context of climate change is effective in promoting mitigation behaviors, including actual and/or costly behaviors, the mechanisms through which perspective-taking works, and if the distance to the person adversely affected by climate change matters for the effect. We conducted an online experiment with a non-student sample from Germany (*n* = 557), utilizing a 2 × 2 factorial design, to investigate the impact of perspective-taking and distance on three outcome measures: a climate donation, signing a petition, and approval of mitigation policies. We find that perspective-taking does not promote these mitigation behaviors, yet it raises the degree perspective-takers value and – for close others – feel connected with the affected person. Exploratory analysis shows that dispositional perspective-taking and empathic concern are correlated with mitigation behaviors.

## 1. Introduction

Taking the perspective of another has been shown to facilitate pro-social behavior (Batson, 1991, 2011). Various studies indicate that this might also apply in the environmental context: research from both social/environmental psychology and experimental economics found positive effects of perspective-taking on pro-environmental behavior or indicators like pro-environmental intentions (see Heinz and Koessler, 2021 for a review). Could perspective-taking thus be used as a complement to standard environmental policy instruments to address global environmental challenges like climate change? The literature has so far either investigated the effect on hypothetical or low-cost behaviors (e.g., Pahl and Bauer, 2013; Pfattheicher et al., 2016), while mitigating climate change requires actual and costly behavioral change. Studies employing such actual or costly behavioral measures have examined behavior only in the context of local environmental problems with identifiable cause-effect structures, such as water use along rivers (Czap et al., 2015; Ortiz-Riomalo et al., 2021). Thus, in this paper, as a first contribution to the literature, we ask whether perspective-taking has the potential to promote actual and/or costly pro-environmental behavior in the context of a more complex and global environmental problem, i.e., climate change.

To assess this potential, it is vital to understand how and in which situations perspective-taking can promote pro-environmental behavior. This implies a need to understand whether the same mechanisms by which perspective-taking has been identified as conducive to pro-social behavior (Batson, 1991; Cialdini et al., 1997) also apply to pro-environmental behavior, where effects on others are less clearly linked to one’s own behavior. Moreover, for climate change in particular, negative consequences are largely borne by people distant from those who have the greatest mitigation leverage (IPCC, 2018; Hickel, 2020). Yet, a common reproach against using empathy-inducing methods is that their effectiveness may be limited to close others (Bloom, 2017). This could imply that the effect of perspective-taking, closely intertwined with empathy (Batson, 2011), is dampened with increasing distance.

Hence, as a second contribution to the existing literature, we examine the *mechanisms* through which perspective-taking works in our environmental context, and, as a third contribution, whether distance to the person affected by climate change moderates the effect of perspective-taking.

To this end, we conducted an online experiment in which we altered perspective-taking: we induced participants to take the perspective of a person affected by climate change caused floods vs. asked them to stay objective; second, we altered who was affected by the floods: a person in Germany vs. a person in India. We measured several observed and/or costly mitigation behaviors and possible mediators.

The remaining paper is structured as follows: The next section gives an overview of the literature on perspective-taking and pro-environmental behavior. Thereafter, we provide the details of our study including sample and materials. Then, we present our study results, discuss them by linking them to existing theory and pointing to their limitations before we conclude by drawing implications for future research and policy.

### 1.1. Perspective-taking and pro-environmental behavior: evidence and hypotheses

Perspective-taking refers to the *“active cognitive process of imagining the world from another’s vantage point or imagining oneself in another’s shoes to understand their visual viewpoint, thoughts, motivations, intentions, and/or emotions”* (Ku et al., 2015, p: 79). This can be a conscious or unconscious effort (Hodges et al., 2011). Thus, the effort can (at least partially) be influenced, and perspective-taking may be externally induced.

There is ample empirical evidence that perspective-taking with a person in need can foster pro-social behavior, ranging from offering volunteering work to taking electric shocks in place of someone else (for an overview, Batson, 1991, 2011). Moreover, perspective-taking may help overcome social divides: many studies show it can reduce stereotypes and prejudice toward marginalized groups (e.g., Dovidio et al., 2004; Wiese et al., 2018). The link between perspective-taking and pro-environmental behavior is less scientifically explored, but first studies originating from different behavioral disciplines have opened this interesting research field.

### 1.2. Perspective-taking as a mean to promote pro-environmental behavior

Pro-environmental behavior refers to a range of different behaviors that have a positive impact on the environment, ranging from consumption to activism and other public sphere behaviors to behaviors in organizations (Stern, 2000). As environmental conditions are closely linked to human well-being (Millennium Ecosystem Assessment, 2005), one’s pro-environmental behavior also has a positive impact on other people. Thus, pro-environmental behavior can be seen as a special type of pro-social behavior, which involves foregoing personal benefits or accepting additional costs or inconveniences for the benefit of others.

Experimental studies from social/environmental psychology and experimental economics provide evidence that perspective-taking can effectively promote pro-environmental behaviors or proxy indicators like intentions (for an overview, Heinz and Koessler, 2021). The review paper by Heinz and Koessler (2021) synthesized the experimental findings on the potential to promote PEB by addressing other-regarding preferences and found that, besides providing information on behavioral consequences, direct appeals, issue framing, moral recategorization, and perspective-taking proved to be an effective approach in this respect. The reviewed psychological experiments report a positive effect by inducing perspective-taking via respective instructions (e.g., “try to feel what the other feels”) vs. the instruction to stay neutral (“try to stay as neutral and objective as possible”). These studies report a positive effect on pro-environmental behavior after taking the perspective of other humans (Pahl and Bauer, 2013; Pfattheicher et al., 2016), animals (Shelton and Rogers, 1981; Schultz, 2000; Berenguer, 2007), or plants (Berenguer, 2007) negatively affected by environmental degradation. The only study that did not find an effect on pro-environmental decision-making is Berenguer (2010). While in their study, a behavioral effect was absent, perspective-taking did increase the number of moral arguments participants named for environmental protection.

All psychological studies mentioned above are lab studies with student samples and relatively low sample sizes. The dependent variables aimed at capturing pro-environmental behavior were generally proxies like intentions (e.g., Pfattheicher et al., 2016) or hypothetical decisions (e.g., Berenguer, 2007). In research, however, it is well established that a gap exists between hypothetical and actual behavior (e.g., Diekmann and Preisendörfer, 2003). Only Pahl and Bauer (2013) examined actual behavior in the form of time spent looking at pro-environmental information material and the number of brochures collected. While providing initial evidence that perspective-taking can influence actual behavior, further research is warranted to assess whether it also has an impact on other costly behaviors.

First insights in this regard are provided by the experimental economic studies of Ortiz-Riomalo et al. (2021) and Czap et al. (2012, 2015). All three studies, following the principle of incentivization, use costly behaviors as outcome measures and report positive effects of perspective-taking. Ortiz-Riomalo et al. (2021) conducted a lab-in-the-field experiment in a Peruvian watershed, where downstream farmers benefitted in water quantity and quality from the traditional and sustainable farming practices of upstream farmers. Once downstream farmers were induced to take the perspective of these upstream farmers, they were more willing to give up financial resources to improve the livelihoods of the upstream farmers.

Also, in the context of water conservation, Czap et al. (2015) gave participants the role of up- and downstream farmers in a framed lab experiment. Upstream farmers could choose between intensive or conservation tillage, and affected downstream farmers could send messages to their upstream fellows. When affected downstream farmers requested upstream farmers to take their perspective and “walk in their shoes,” upstream farmers were less likely to drop their conservation efforts when a previously installed pecuniary compensation was removed. For the same game, Czap et al. (2012) found positive effects on conservation decisions of priming participants to take the perspective of downstream farmers. Although all decisions in these experiments involved pecuniary costs, the perspective-taking results may be limited to the specific situation of local environmental interactions. In our study, we assess whether the positive effect of perspective-taking also holds for costly behaviors in the context of a more complex and global environmental problem, i.e., climate change.

Given the positive associations of perspective-taking and pro-environmental behaviors found in previous studies, our first hypothesis is,

*H*1: Individuals who are induced to take the perspective of people negatively affected by climate change are more willing to engage in mitigation behaviors than individuals who are asked to stay objective.

### 1.3. Through which channels does perspective-taking work in an environmental context?

In the literature, different views exist on how perspective-taking unfolds its positive effect on general pro-social behavior (Hodges et al., 2011; Sassenrath et al., 2022). The most prominent scientific debate discusses whether the pro-social effect of perspective-taking is motivated by altruistic or egoistic reasons. According to Batson (1991, 2011), perspective-taking raises empathic concern with others, which instills an altruistic motivation to act. More precisely, valuing the others and perceiving their need are the two antecedents of empathic concern. Both have been shown to be addressed by perspective-taking (Batson, 1991, 2011).

A different explanation is given by Cialdini et al., who theorize that perspective-taking leads to a merging between the self and the other, in other words, an increase in oneness (Cialdini et al., 1997; Maner et al., 2002). By this mechanism, they argue, pro-social behavior resulting from perspective-taking has an inherent egoistic component. Also here, empirical studies provide supportive evidence that oneness increases through perspective-taking with another being (e.g., Cialdini et al., 1997; Galinsky and Moskowitz, 2000). The literature also shows that this merging between the self and the other is two-directional: others are seen as more self-like and the self is seen as more other-like (for an overview see Galinsky et al., 2005; Hodges et al., 2011). While it is debatable if an increased perception of oneness really means that pro-social behavior is egoistically motivated, it certainly shows that perspective-taking makes the relationship between perspective-taker and perspective-giver closer: it connects them.

So far, the empirical evidence lends support to both mechanisms to be relevant mediators (Maner et al., 2002; Hodges et al., 2011). Hitherto, in the context of pro-environmental behavior, these mediation pathways still need to be explored. In our study, we investigate both theoretical underpinnings for an effect of perspective-taking in the environmental context. Consequently, our second hypothesis is,

*H*2a–c: Individuals who are induced to take the perspective of people negatively affected by climate change show greater levels of (a) perception of need, (b) valuing of the other, and (c) oneness, compared to individuals who are induced to stay objective.

### 1.4. Does the distance to the affected parties matter for the effect?

To judge the usefulness of perspective-taking as a means to promote mitigation behaviors, it is important to understand whether its effect depends on whose perspective is taken. For instance, existing evidence suggests that the effect of perspective-taking on action and its ability to evoke empathic concern may hinge on the personal and social characteristics of the perspective-giver, which in turn influences the perspective-taker’s valuation of his or her person (Batson, 2011). For instance, perceived similarity (e.g., Batson et al., 1995) or social systems of devaluation of people (like racism; Dietz et al., 2018) may determine to what degree value is given to the perspective-giver. In the literature, this limited scope of empathic concern is a common criticism directed toward empathy-based approaches as political instruments (Bloom, 2017; Breithaupt, 2017).

For the environmental context, the most interesting is the question whether the effect of perspective-taking depends on who is affected by environmental degradation. In the case of climate change, adverse consequences have to be endured mostly by people who are somehow “distant” from those with the greatest mitigation leverage: while the largest share of greenhouse gas emissions stems from operations in the Global North (Hickel, 2020), the most severe consequences of climate change are felt by people living in the Global South (Mendelsohn et al., 2006; IPCC, 2018). For instance, although flood risk is also increasing in Germany due to climate change (Te Linde et al., 2011; Sairam et al., 2021), the greatest risk is predicted for Southeast Asia, India, eastern Africa as well as parts of the Andean region (Hirabayashi et al., 2013; Rentschler et al., 2022). Thus, mitigating climate pressure from people most affected by climate change will require an enormous collective behavioral change from people who are distant from them in terms of residence and cultural background. We have shown in Heinz et al. (2023) that distance to those affected by climate change can indeed decrease the willingness to carry out (low-cost) mitigation. In the present paper, we examine how distance interacts with perspective-taking. The direction of the interaction is unclear. On the one hand, distance could lower the effect of perspective-taking because it is limited to close others, as claimed by its critics (Bloom, 2017; Breithaupt, 2017). Perspective-taking with people far from oneself requires the activation of higher-order cognitive skills (Hoffman, 2000). When the perspective-giver is far, perspective-taking may be more difficult. Based on these considerations, we would expect perspective-taking with people far from the perspective-taker to be *less* effective in promoting mitigation behaviors than when the perspective of close others is taken. Yet, the opposite might also be true. In fact, perspective-taking may help to bridge the gap, i.e., it may reduce distance and its effect on decision-making (Liberman and Trope, 2014; *cf.* Pahl and Bauer, 2013). According to Batson (2011), perspective-taking with close, i.e., valued, others happens automatically. This means that intentionally adopting someone’s perspective might actually exert a stronger influence when performed with people with whom perspective-taking does not automatically take place – i.e. distant others. Hence, *externally induced* perspective-taking could also be *more* effective in increasing mitigation behaviors when it is directed toward distant others. In addition, it may be the case that no interaction takes place, either because both effects offset each other, resulting in an overall null effect, or because there simply is no moderating effect of distance on perspective-taking.

In our study, we test whether the effect of perspective-taking on mitigation behaviors depends on distance by offering the perspective of a person affected by climate change induced floods who reportedly lives either in Germany (CLOSE) or in India (FAR). As a testable hypothesis, we predict to find an interaction effect without defining its direction.

*H*3a-b: Distance between perspective-taker and perspective-giver moderates the effect of perspective-taking on the willingness to engage in (a) costly mitigation behaviors and (b) the mediation pathways.

## 2. Methods

### 2.1. Participants and design

#### 2.1.1. Overview

The data was collected in an online experiment with a sample of 557 non-student participants (see Table 1).^1^ Participants were randomly allocated across treatment conditions: One treatment variation induced either perspective-taking with a person negatively affected by climate change or an objective mindset (PERSPECTIVE-TAKING vs. STAY OBJECTIVE). The other treatment variation altered who was negatively affected (CLOSE: a person in Germany vs. FAR: a person in India). Afterward, various variables of mitigation behaviors and possible mediators were elicited before participants were asked for sociodemographic characteristics, own flooding experience, and migration background as control variables. The study design was ethically approved by the LaER Ethics Committee of Osnabrück University.

**Table 1:** Overview of experimental design and observations.

	STAY OBJECTIVE	PERSPECTIVE-TAKING
CLOSE (Paul Weber, Germany)	A (*n* = 136)	B (*n* = 141)
FAR (Samudra Sudarshan, India)	C (*n* = 132)	D (*n* = 148)

#### 2.1.2. Participants

Participants were recruited from the online platform *clickworker* to take part in an online survey about the “consequences of climate change.” They were paid a remuneration of 10€, of which they could donate 0–5€ (one of our dependent variables). Hence, all participants ended up with a payment between 5 and 10€. The average duration to fill out the survey was approximately 25 min.

#### 2.1.3. Data exclusion

We excluded data from (i) participants with very short total answering time as we can assume that these participants did not take the task seriously. We define very short total answering times as being less than half of the average total answering time. We further excluded data from (ii) participants who failed both attention checks and from (iii) participants who responded with “always” or “sometimes” to an item at the end of the questionnaire asking, whether they gave meaningless responses. Finally, we excluded data from (iv) participants who did not finish the study. After data exclusion, we checked whether randomization was still successful in terms of the distribution of the sociodemographic variables (income, education, gender, and age) and further control variables relevant to our research question (flooding experience and migration background). Attributes were evenly distributed except gender and flooding experience (see Supplementary material Table A1). To account for potential effects stemming from these characteristics, we control for these variables in our regression analysis.

#### 2.1.4. Sample

All participants lived in Germany and spoke German as native language (our filter configurations to keep distance constant). 16% reported having a migration history in their family, while none of them had parents or grandparents from India in specific. 16.3% indicated that they had experienced flooding themselves. The sample consisted of 239 females, 314 males, two people identifying as diverse and two people without specification. Age ranged from 18 to 74 years, with a mean age of 34.4 years old. Almost half (43.8%) of the study participants had a university degree. In our regression models, we account for this set of sociodemographic variables to probe the robustness of our results.

#### 2.1.5. Time

The experiment took place in April 2020. Thus, it was carried out during the first peak of the COVID-19 pandemic. We discuss later how this might have impacted our results.

### 2.2. Procedure and treatments

#### 2.2.1. Procedure

At the start, all participants were introduced to the basics of climate change and how it increases the risk and intensity of floods. They were asked two multiple-choice questions after reading the text for an attention check. Then, an example was provided of how people experience extreme flooding events: Participants read an interview of a person who recounted how their house was flooded. Interview questions were about how the persons experienced the flooding event, how it felt when they came back to the house and how the flooding changed their attitude toward life. The interview was composed of original statements from people who had experienced floods (Interview text and the treatment variations can be accessed in Online material).^2^

#### 2.2.2. Perspective-taking treatment

The first treatment alteration consisted of inducing perspective-taking with the person negatively affected by climate change vs. a neutral mindset. Before reading an interview with the affected person, participants were given varying instructions. In the PERSPECTIVE-TAKING condition, participants were given the instruction to adopt the perspective of the interviewed person and concentrate on their feelings and thoughts. In the STAY OBJECTIVE condition, participants were asked to stay objective and take a neutral perspective, just concentrating on the described facts. Such variations of instructions have been used successfully in various experiments to increase low-cost pro-environmental behaviors or indicators thereof (Shelton and Rogers, 1981; Berenguer, 2007; Pahl and Bauer, 2013; Pfattheicher et al., 2016). Previous research has also shown that altering instructions has a physiologically measurable effect on empathic emotional arousal (Stotland, 1969; Lamm et al., 2007). In order to strengthen our manipulation, we included an interactive task after the interview. In the PERSPECTIVE-TAKING condition, participants were asked to write a letter to the person as a friend. In the STAY OBJECTIVE condition, participants were asked to write a neutral report of the event as a journalist. For both tasks, a minimum of 300 characters were required to continue the survey.

#### 2.2.3. Distance treatment

The second treatment alteration, aimed to test H3, manipulated *who* was negatively affected by climate change. In the CLOSE condition, the person giving the interview was named Paul Weber, living in Rhüden, a small town in central Germany. In the FAR condition, the person was named Samudra Sudarshan, living in Hatipara, an equally sized town in eastern India. So, a person that is culturally and physically distant to the participant. A big map was shown before the interview to visually demark the residence. Additionally, a small map was presented above the interview text to keep residence salient, and questions always referred to Paul Weber or Samudra Sudarshan, respectively.

### 2.3. Manipulation checks

#### 2.3.1. Manipulation check – perspective-taking treatment

To check if our perspective-taking treatment was successful in inducing the intended state of mind, we employed two manipulation check items, which have been used in a similar form by Batson in various studies (e.g., Batson et al., 2002). After reading the interview, participants were asked to state to what extent they took the perspective of the other person and to what extent they stayed objective (1 = not at all, 7 = fully). The second item was reverse coded and the average of both items was taken as an indicator for the level of (self-reported) perspective-taking. Comparing indicator values between the STAY OBJECTIVE and PERSPECTIVE-TAKING treatment groups with a Mann–Whitney-U-test, we find the difference to be significant with *p* < 0.000. As expected, the average is higher in the PERSPECTIVE-TAKING condition (M = 5.07) compared to the STAY OBJECTIVE condition (M = 4.02).

#### 2.3.2. Manipulation check – distance treatment

To assess the saliency of the second treatment, we asked participants after completing the entire survey about the name and residence of the interviewed person, i.e., Paul Weber in Germany in the CLOSE condition or Samudra Sudarshan in India in the FAR condition. Well above 95% of study participants correctly identified the name and residency of the interviewed person in the respective treatment group (correct answers for Paul Weber: 99.6%, Germany: 98.9%; Samudra Sudarshan: 99.3%, India: 95.4% – we provide the full details in the Supplementary material). That is, our treatments were successful: it was salient to the study participants who were affected by climate change.

### 2.4. Outcome measures: mitigation behaviors

In our study, we are interested in actual and/or costly mitigation behaviors. All behaviors are aimed at reducing greenhouse gas emissions and thus have a positive impact on climate protection. With the help of three variables, we capture participants’ willingness to engage in mitigation behaviors: (1) willingness to donate money to a mitigation NGO, (2) willingness to sign a petition, and (3) willingness to approve mitigation policies (Supplementary Online material provides the exact description of how these variables were elicited. Supplementary material Table A2 shows weak positive correlations between the mitigation behaviors.).

#### 2.4.1. Donation

Our first dependent variable is an actual donation made to a climate NGO, with which we capture participants’ willingness to give up own financial resources. Specifically, we asked participants at the end of the survey if they wanted to donate 0–5€ (in steps of 0.5€) of their 10€ remuneration to the pro-environmental NGO *atmosfair*, which finances climate protection projects all over the world to compensate for CO_2_ emissions. Participants were told that *atmosfair* operates with the highest standard for CO_2_ emissions reduction projects (CDM Gold Standard) and that a donation of 1€ equals an approximate reduction of 40 kg of CO_2_. We chose *atmosfair* because the climate benefits of CO_2_ compensation can be felt globally and thus benefit a person in Germany and India to the same extent. The total amount donated by the study participants was transferred to *atmosfair* after the study.

#### 2.4.2. Petition

As our second dependent variable, we elicited the willingness to sign a petition in the context of climate change mitigation as a means to engage in environmental citizenship behavior (Dobson and Bell, 2006; *cf.* Stern, 2000). Participants were asked if they wanted to receive a link for signing a petition for more climate protection in Germany and could leave their email address for that purpose (which was stored separately from the rest of the dataset to secure anonymity). While not involving pecuniary costs, this variable was observed and costly in the sense that people had to provide personal data and dedicate time. Participants who agreed received the link after the study; yet we do not know whether they actually signed the petition. We use the willingness to provide the email address (yes/no) as a proxy for the willingness to sign a petition.

#### 2.4.3. Policy approval

As our third dependent variable, we elicited whether people were willing to approve structural changes in favor of climate protection at their own cost or discomfort. Participants were presented with a set of 12 political measures which, at the time, were discussed in the political debate to mitigate climate change. These included command and control instruments (e.g., ban of domestic flights), price-based instruments (e.g., introduction of a CO_2_ tax) as well as more ambitious political goals (e.g., phasing out from coal power until 2030 instead of 2038). Study participants could answer on a 5-point scale whether they personally fully approved (+2) to fully disapproved (−2) an introduction of the respective measure. The average rate of approval (Cronbach’s α =0.856) served as the final dependent variable, i.e., the willingness to approve the costly political measures.

### 2.5. Mediation pathways

#### 2.5.1. Perception of need/valuing of the other

To test Batson’s theory that perspective-taking raises empathic concern with others with the two antecedents “valuing the other” and “perceiving his or her need,” we used two items similar to those from Batson et al. (2002). One item asked participants to which extent they perceived the situation of Paul Weber/Samudra Sudarshan as an emergency (perception of need). The other item asked how much they cared for Paul Weber’s/Samudra Sudarshan’s well-being (valuing the other). Both items could be answered on a 7-point scale (1 = not at all, 7 = fully).

#### 2.5.2. Oneness

To assess the explanatory power of Cialdini’s theory that the effect of perspective-taking stems from a merging between the self and the other, we used the Inclusion of Other in Self (IOS) scale developed by Aron et al. (1992) to measure perceived oneness between perspective-taker and perspective-giver. Participants were shown seven pictures of two circles representing themselves and Paul Weber/Samudra Sudarshan with varying distances or degrees of overlap and were asked to select the depiction that best described their perceived closeness to the other person.

## 3. Results

**Figure 1:**
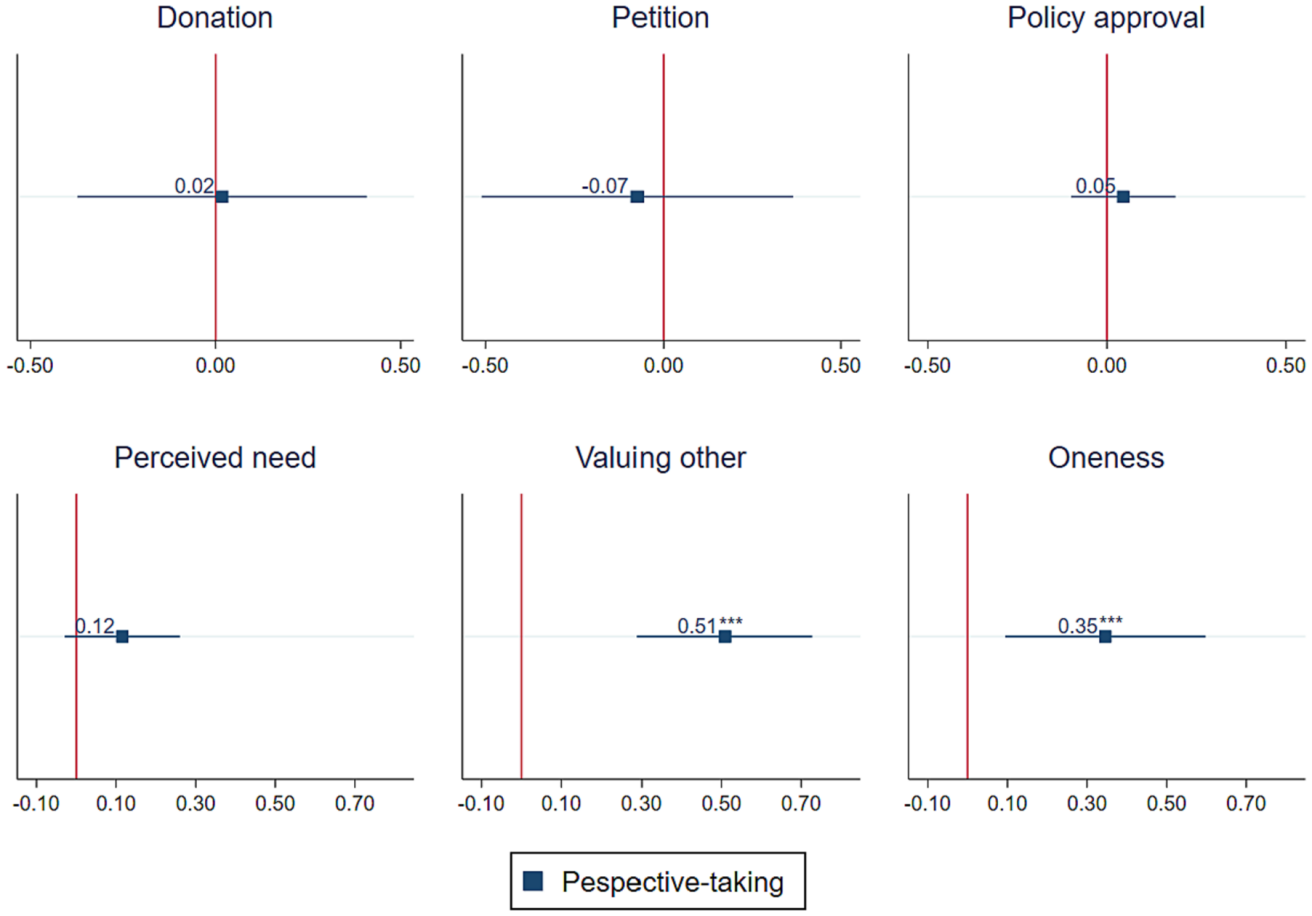
Effect of perspective-taking on mitigation behaviors and mediators. The figure shows the point estimates (squares) and 95% confidence intervals (lines) of the respective regression models for the treatment dummy “PERSPECTIVE-TAKING.” The symbols *, **, and *** indicate significance at *p* < 0.05, *p* < 0.01, and *p* < 0.001, respectively.

To test whether perspective-taking leads to a higher willingness to engage in mitigation behaviors (H1), we performed Chi2 tests for the donation and the petition as well as a Mann–Whitney-U test for policy approval, comparing the respective behaviors in the PERSPECTIVE-TAKING vs. STAY OBJECTIVE condition (pooling the data for CLOSE and FAR). For none of the three mitigation behaviors, we find significant differences between the two treatment conditions [donation: χ^2^(1) =0.067, *p* = 0.796; petition: χ^2^(1) = 0.085, *p* = 0.771; and policy approval: z = −0.905, *p* = 0.365]. The same result is obtained when running regression analysis that accounts for the influence of potential covariates by including control variables (socio-demographic characteristics as well as own flood experience and migration background; logit models for the donation and the petition, ordinary least square regression models for policy approval). Results of the regression analysis are depicted graphically in the upper row of Figure 1, which shows, for each dependent variable, the regression coefficients and corresponding confidence intervals for the treatment dummy “PERSPECTIVE-TAKING.” The respective regression tables can be found in the Supplementary material Table A3. In sum, our perspective-taking intervention did *not* increase participants’ willingness to engage in costly mitigation behaviors. Thus, we do not find support for our first hypothesis.

For our second hypothesis (H2), we aimed to test the mediation channels as proposed by Batson (2011) – (a) perception of need and (b) valuing of the other and Cialdini et al. (1997) – (c) oneness; 1997. Although we did not find effects on the behavioral variables, our treatments could have exerted an effect on these channels without translating into action. Estimating the treatment effects with help of ordinary least square regression models, while accounting for the control variables (see Supplementary material Table A4), we find that perspective-taking did increase the valuing of the other (*p* = 0.000) as well as oneness (*p* = 0.007). For the perception of need, the likewise positive effect of perspective-taking failed to be statistically significant (*p* = 0.11). The bottom row of Figure 1 depicts again graphically the coefficients and confidence intervals for the treatment dummy “PERSPECTIVE-TAKING.” In sum, our findings are supportive of our hypotheses 2b and 2c: Individuals induced to take the perspective of people negatively affected by climate change show higher levels of valuing of the other and oneness, compared to individuals who were induced to stay objective. We do not find evidence for hypothesis 2a as the impact of perspective-taking on the perception of need is non-significant.

**Figure 2:**
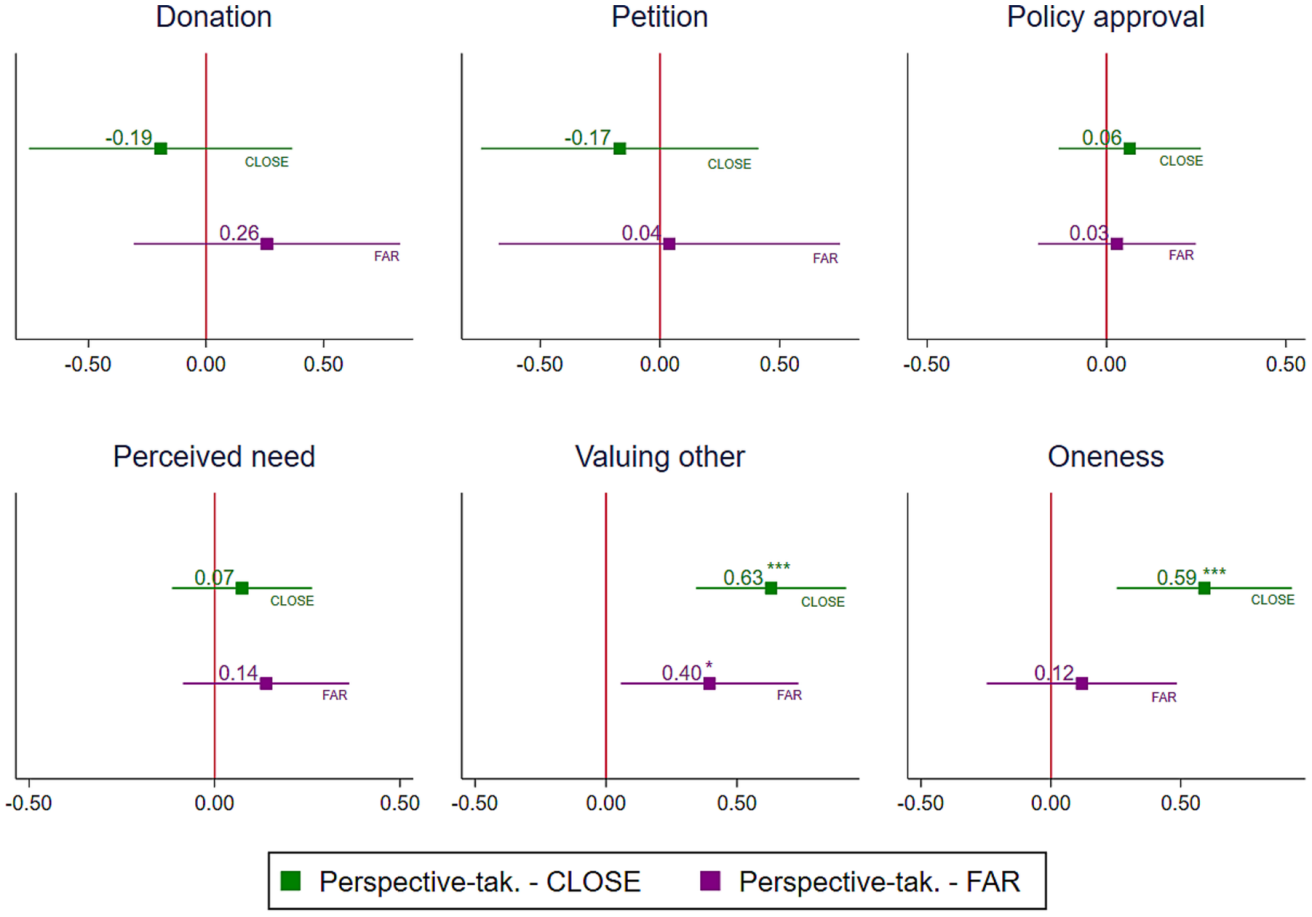
Effect of perspective-taking on mitigation behaviors and mediators split for the CLOSE and the FAR treatment. The figure shows the point estimates (squares) and 95% confidence intervals (horizontal lines) of the estimated effects of PERSPECTIVE-TAKING in the two subsamples CLOSE and FAR. The symbols *, **, and *** indicate significance at *p* < 0.05, *p* < 0.01, and *p* < 0.001, respectively.

Lastly, we assess whether the distance between the perspective-taker and the perspective-giver, i.e., the person affected by climate change, moderates the effect of perspective-taking on mitigation behavior (H3a) and on the mediation pathways (H3b). For this purpose, we estimated the effect of perspective-taking for our CLOSE and FAR treatments separately, with the help of regression models. Figure 2 displays the estimated treatment effects for each subgroup. Supplementary material Tables A5, A7 show the corresponding numerical regression results for mitigation behavior and mediation pathways, respectively. Supplementary material Table A5 shows that in both subsamples, CLOSE and FAR, perspective-taking has no significant effect on the three outcome variables with which we measured mitigation efforts. An equality test on the coefficients for “PERSPECTIVE-TAKING” between the two subsamples reveals in addition that no structural difference exists in the impact perspective-taking unfolds on the mitigation behaviors in the CLOSE vs. FAR condition [donation: χ^2^(1) = 1.24, *p* = 0.266, petition: χ^2^(1) = 0.19, and policy approval: χ^2^(1) = 0.06, *p* = 0.663, and *p* = 0.810]. This means we do not find support for hypothesis H3a – there was no difference in the effect of perspective-taking on the mitigation behaviors depending on who was affected by the floods (Paul Weber in Germany or Samudra Sudarshan in India).

For the mediators, results are shown in the lower part of Figure 2, based on the regression results in Supplementary material Table A7. For the “perception of need,” perspective-taking failed to produce a significant effect in both subsamples, and no structural difference was found in its effect on perceived need between CLOSE vs. FAR (*p* = 0.666). For “valuing the other,” the effect of perspective-taking is statistically significant for both conditions, with *p* = 0.000 for CLOSE and *p* = 0.022 for FAR. However, also here, no significant structural difference exists in the effect perspective-taking has in the two subsamples (*p* = 0.288). For the feeling of oneness, perspective-taking had a significant influence in the CLOSE (*p* = 0.001) but not in the FAR treatment condition (*p* = 0.522). Here, the performance of the equality test on the coefficients for “PERSPECTIVE-TAKING” indicates that a structural difference between the two distance conditions might exist, but statistical significance is at the margin of conventional significance levels [χ^2^(1) = 3.60, *p* = 0.058]. Thus, in sum, for the perception of need and valuing of the other (H3a and H3b), we do not find evidence for a moderation effect of distance. For the perception of oneness, we find some indication that distance might moderate the effect of perspective-taking (H3c).

## 4. Discussion

In our experiment, perspective-taking did not increase the willingness to engage in mitigation behaviors and there was no difference in this (lack of) effect depending on who was affected by climate change. How can it be explained that our results differ from previous studies that found significant positive effects of perspective-taking on the engagement in pro-environmental behaviors (e.g., Berenguer, 2007; Pfattheicher et al., 2016)? As we laid out in the beginning, we are looking at a selection of actual and/or costly behaviors, while the other studies conducted in the context of climate change examined hypothetical or low-cost behaviors. Hence, it seems plausible that perspective-taking may promote pro-environmental cognitions, emotions, and intentions, but our results cast doubt on whether it is sufficient to invoke actual and/or costly behavior. With this finding, we would like to encourage future research which tests the effect of perspective-taking also for other forms of costly environmentally relevant behaviors in which individuals can engage in (Nielsen et al., 2021), such as consumption or investment decisions.

As a second contribution, we examined behavior in the context of climate change, which inherently constitutes more complex environmental context than the local environmental problem of water conservation, within which the effect of perspective-taking had been analyzed in previous studies. Not only are the causes of climate change more difficult to disentangle, but also finding and implementing solutions to the problem encompasses a multitude of activities and stakeholders. Hence, self-efficacy is limited and individuals may feel that the impact of their pro-environmental actions is negligible (*cf.* Value-Belief-Norm Theory; Stern et al., 1999). This may explain why perspective-taking was successful in previous studies focused on promoting costly pro-environmental behaviors in local settings (Czap et al., 2015; Ortiz-Riomalo et al., 2021) but not in our study. This explanation may be supported by the fact that studies on general prosocial behavior, which reported significant positive effects of perspective-taking, were based on more direct forms of pro-social behavior like providing assistance, for which a higher (self-) efficacy can again be assumed compared to our case (Batson, 1991, 2011).

Regarding the underlying mechanisms of why and how perspective-taking works, our findings lend some support to both Batson’s and Cialdini’s explanations: valuing the other and perceived oneness were higher in the perspective-taking condition than in the stay objective condition. Our results also show that oneness and valuing the other are highly correlated (Spearman’s rho = 0.437, *p* = 0.000). Experimental research and a meta-analysis by McAuliffe et al. (2018, 2020, respectively) suggest that differences found in perspective-taking vs. stay objective conditions may not stem from a positive effect of the perspective-taking instructions on empathic concern, as commonly assumed, but from a reducing effect of the stay objective instructions. While we do not have the design to single out the effect of the two instructions, McAuliffe’s research indicates that people’s inclination to take the perspective of a person in need can be actively suppressed. Their findings also support Batson’s empathy-altruism hypothesis: the effects of the two conditions on their outcome variable of social support seem to be mediated by changes in empathic concern and not through an altered perception of oneness (McAuliffe et al., 2018). McAuliffe and colleagues reached this conclusion by examining the effects of different instructions on empathic concern. In our own experiment, the complementary reinforcing task (writing a letter as a friend vs. writing a neutral newspaper article) may have prompted participants to engage with the interviewed person’s experience in both conditions, thereby possibly overriding the suppression effect of the stay-objective condition and ultimately resulting in the null result on the behavioral measures.

In their meta-study, McAuliffe et al. (2020) also examined the possible moderating effect of outgroup membership of the perspective-giver, but the results were inconclusive. Our experimental results suggest that there is a difference in how perspective-taking works depending on whose perspective is taken. Specifically, distance seemed to moderate the effect of perspective-taking on oneness: perspective-taking only unfolded a statistically significant effect when a person in Germany was affected, but not when a person in India, who is culturally and physically distant, was affected. Perspective-taking was able to narrow the divide between the self and the other only for close others. In that respect, the reproach made against empathy-inducing approaches seems justified in the sense that they may discriminate between different groups of people. However, perspective-taking did increase the valuing of the other for both close and far people. Yet, we also found that this was not enough to endure costs to benefit those others.

To determine whether empathy and perspective-taking played in our experiment any role at all, we conducted an additional exploratory analysis (reported in Supplementary material Table A8). We focused hereby on dispositional empathic concern and perspective-taking – measured by the Interpersonal Reactivity Index (IRI; Davis, 1980, subscales following Cliffordson, 2001) – as general and more stable personality traits. Using questions such as “When I see someone being taken advantage of, I feel kind of protective towards them” (empathic concern) or “I sometimes try to understand my friends better by imagining how things look from their perspective” (perspective-taking), the IRI elicits a person’s inherent likelihood to engage with the emotional and cognitive aspects of empathy. For our experiment, we find that dispositional empathic concern is indeed predictive of the willingness to engage in mitigation behaviors (donation *p* = 0.011, petition *p* = 0.001, and policy approval *p* = 0.000). For the cognitive aspect of empathy, i.e., the ability to take another’s perspective, we find that it influences the willingness to sign a petition (*p* = 0.046) and to support structural change through policy approval (*p* = 0.000), but is not associated with the donation, the mitigation behavior involving direct pecuniary costs. Thus, a connection between perspective-taking/empathy and the willingness to undertake mitigation efforts also exists in our experiment, yet the incremental change from the induced perspective-taking appears not to have been strong enough to alter behavior further. This can also be interpreted as being in line with the empathy-altruism hypothesis and the findings of McAuliffe and colleagues.

Lastly, it must be mentioned that our study was conducted at the beginning of the COVID-19 pandemic, which may have affected our results. In fact, a pretest that we ran before the main data collection indicated results more consistent with H1 and H3, but the sample was too small to take this as a solid finding. It is possible that people experienced something like a perspective-taking overload because they were confronted with extensive suffering during this time. Also, the crisis may have strengthened the focus on self-protection. Thus, people might have been less receptive to the perspective-taking intervention. A study by Todd et al. (2015) supports this possible caveat. Their study experimentally varied the level of anxiety in participants and found that more anxious states negatively interfered with the perspective-taking capacities of people (in line with their general finding that anxiety promotes self-centeredness). Thus, the null effect of perspective-taking we found in our experiment may have also been shaped by the extraordinary stress induced by a global crisis.

## 5. Conclusion

In this paper, we experimentally tested whether perspective-taking with someone negatively affected by climate change increases the willingness to engage in actual and/or costly mitigation behaviors. Moreover, we asked through which mechanisms such an effect may take place and whether the results depend on who was negatively affected. We found that perspective-taking did not increase mitigation behaviors. Results did not differ depending on whether the person affected lived in Germany or India. For the mechanisms, our results show that perspective-taking increased the valuing of the other person and the perception of oneness. These results, contrary to the behavior, varied with who the perspective-giver was: only for the person in Germany did the induced perspective-taking narrow the perceived divide between the self and the other.

Future research should continue to investigate the effect of perspective-taking on actual and costly pro-environmental and mitigation behaviors, for instance, by applying a similar design in a time without an acute crisis or by designing stronger perspective-taking interventions (e.g., multiple interventions or with tasks that require more active perspective-taking). Our study provides a starting point for future research to investigate the different functioning of perspective-taking depending on whose perspective and under which conditions it is taken in the environmental context.

In terms of policy implications, our results give reason to question the use of perspective-taking as a policy approach in a global environmental context for inducing actual and costly behavioral change. Our exploratory analysis implies, however, that dispositional perspective-taking and empathy go in hand with an increased willingness to take action and support structural change for climate protection. Consequently, a more constant cultivation of both attributes could possibly help to lay the ground for a democratically legitimized sustainability transformation.

## Data availability statement

The raw data supporting the conclusions of this article will be made available by the authors, without undue reservation.

## Ethics statement

The studies involving humans were approved by LaER Ethics Board, Osnabrück University. The studies were conducted in accordance with the local legislation and institutional requirements. The participants provided their written informed consent to participate in this study.

## Author contributions

A-KK, NH, and SE contributed to conception and design of the study. NH organized the data collection and wrote the first draft of the manuscript. A-KK performed the statistical analysis and undertook the final write up. All authors contributed to the article and approved the submitted version.

## Footnotes

^1^The present paper builds on our previous work. For the control treatments STAY OBJECTIVE CLOSE and STAY OBJECTIVE FAR, we report data from Heinz et al. (2023). Data for PERSPECTIVE-TAKING CLOSE and PERSPECTIVE-TAKING FAR is being reported for the first time. All data was collected in April 2020 in one wave.

^2^ https://osf.io/b54z3/?view_only=11f7b16c995f49b6a6b2d6975de9d428
