# Supplementary material for: Perspective-taking with affected others to promote climate change mitigation
Source: Front Psychol. 2023 Sep 28;14:1225165. doi: 10.3389/fpsyg.2023.1225165 (PMC10575756; doi:10.3389/fpsyg.2023.1225165)
Supplement: Supplementary file 1 [file Data_Sheet_1.PDF]

Perspective-Taking with Affected Others to Promote Climate Change Mitigation

Appendix

Manipulation check – Distance treatment

**Condition**	**Correct answers**	**Incorrect answers**
**Close condition**		
Name	Paul Weber (99.6%)	Samuda Sudarshan (0.4%)<br>Simon Derweil (0%)<br>Hussein Malik (0%)
Country	Germany (98.9%)	India (0.7%)<br>Switzerland (0.4%)<br>Iran (0%)
**Far condition**		
Name	Samudra Sudarshan (99.3%)	Hussein Malik (0.7%)<br>Paul Weber (0%)<br>Simon Derweil (0%)
Country	India (95.4%)	Iran (3.2%)<br>Germany (1.4%)<br>Switzerland (0%)

It can be assumed that those participants in the Far condition answering Hussein Malik and Iran also perceived the interviewed person as being socio-spatially from them.

Table A1. Balancing Table.

	(1) Stay objective/CLOSE		(2) Stay objective/FAR		(3) Perspective-tak./CLOSE		(4) Perspective-tak./FAR		t-test Differences					
**Variable**	**N**	**Mean/SE**	**N**	**Mean/SE**	**N**	**Mean/SE**	**N**	**Mean/SE**	**(1)-(2)**	**(1)-(3)**	**(1)-(4)**	**(2)-(3)**	**(2)-(4)**	**(3)-(4)**
**Disposable income (in EUR)**	119	1740.546	109	1755.734	126	1625.992	134	1826.493	-15.188	114.554	-85.946	129.742	-70.759	-200.500
		[98.990]		[102.728]		[96.953]		[98.381]						
**University degree**	136	0.463	132	0.462	140	0.364	147	0.469	0.001	0.099	-0.006	0.098	-0.007	-0.105
		[0.043]		[0.044]		[0.041]		[0.041]						
**Gender (non-male)**	136	0.449	132	0.523	141	0.411	148	0.372	-0.074	0.037	0.077	0.111	0.151*	0.040
		[0.043]		[0.044]		[0.042]		[0.040]						
**Age**	132	36.121	129	33.775	138	33.558	143	34.350	2.346	2.563	1.772	0.217	-0.574	-0.792
		[1.135]		[0.999]		[0.968]		[0.985]						
**Migration background**	134	0.149	127	0.173	139	0.137	146	0.192	-0.024	0.013	-0.043	0.037	-0.019	-0.055
		[0.031]		[0.034]		[0.029]		[0.033]						
**Flooding experience**	136	0.154	131	0.221	141	0.113	147	0.156	-0.067	0.041	-0.002	0.108*	0.065	-0.043
		[0.031]		[0.036]		[0.027]		[0.030]						
**F-test of joint significance (F-stat)**									0.590	1.179	0.526	1.858*	0.969	1.208
**F-test, number of observations**									222	236	242	226	232	246

*Note: The value displayed for t-tests are the differences in the means across the groups. The values displayed for F-tests are the F-statistics. ***, **, and * indicate significance at p<.05, p<.01, and p<.001.*

Table A2. Correlations between dependent variables

**Pearson's Correlation**	PEBgiv_yes	PEBgiv_amount	PEBpet_yes	PEBpol_aver
PEBgiv_yes	1.000	-	-	-
PEBgiv_amount	0.851**	1.000	-	-
PEBpet_yes	0.204**	0.223**	1.000	-
PEBpol_aver	0.234**	0.275**	0.351**	1.000

Note: ** indicates p<0.010

I. ANALYSIS

Table A3. Estimations of the Effect of Perspective-Taking on the Three *Behavioral Measures*

	(1)<br>Donation	(2)<br>Donation	(3)<br>Petition	(4)<br>Petition	(5)<br>Policy approval	(6)<br>Policy approval
**Perspective-taking**	-0.046<br>[-0.401,0.308]	0.018<br>[-0.373,0.409]	-0.058<br>[-0.451,0.335]	-0.073<br>[-0.511,0.365]	0.031<br>[-0.102,0.163]	0.046<br>[-0.100,0.192]
**Disposable income (in EUR)**		-0.00001<br>[-0.000,0.000]		-0.0004**<br>[-0.001,-0.000]		-0.0001***<br>[-0.000,-0.000]
**University degree**		0.420*<br>[0.018,0.822]		-0.025<br>[-0.477,0.428]		0.005<br>[-0.145,0.155]
**Gender (not male)**		-0.030<br>[-0.433,0.372]		0.306<br>[-0.138,0.749]		0.005<br>[-0.145,0.155]
**Age**		-0.006<br>[-0.023,0.011]		0.020*<br>[0.002,0.038]		0.002<br>[-0.005,0.008]
**Flood experience**		-0.261<br>[-0.805,0.282]		-0.094<br>[-0.694,0.506]		-0.075<br>[-0.272,0.121]
**Migration background**		-0.003<br>[-0.539,0.531]		0.012<br>[-0.586,0.609]		-0.027<br>[-0.226,0.173]
**Constant**	-0.699***<br>[-0.953,-0.445]	-0.618<br>[-1.369,0.132]	-1.159***<br>[-1.440,-0.878]	-1.316**<br>[-2.136,-0.496]	0.456***<br>[0.360,0.551]	0.511***<br>[0.233,0.789]
**N**	**557**	**468**	**557**	**468**	**557**	**468**

*Notes: This table shows the estimation results from regressing the impact of the treatment PERSPECTIVE-TAKING on participants' willingness to give up own resources (Donation), give their email address to sign a petition (Petition) and to support structural change (Policy approval). Models (1)–(4) show the coefficients of logit regressions on the likelihood of making a donation or giving their email address to sign a petition. Models (5)–(6) are based on an ordinary least squares regression model that estimates the effect of PERSPECTIVE-TAKING on the average approval of 12 realistic policy measures for climate protection in Germany. 95% confidence intervals are shown in brackets. The symbols *, **, *** indicate significance at p<.05, p<.01, and p<.001, respectively. The coefficient plots at the top of Figure 1 are based on model (2), (4) and (6).*

Table A4. Estimations on the Effect of Perspective-Taking on the *Mediator Variable*

	(7) Perceived need	(8) Perceived need	(9) Valuing other	(10) Valuing other	(11) Oneness	(12) Oneness
**Perspective-taking**	0.121	0.116	0.323**	0.509***	0.238*	0.346**
	[-0.016,0.258]	[-0.029,0.260]	[0.116,0.530]	[0.290,0.728]	[0.010,0.466]	[0.094,0.598]
**Disposable income (in EUR)**		-0.000001		0.00006		0.00009
		[-0.000,0.000]		[-0.000,0.000]		[-0.000,0.000]
**University degree**		-0.024		-0.269*		-0.219
		[-0.173,0.125]		[-0.495,-0.043]		[-0.478,0.041]
**Gender (not male)**		0.159*		0.426***		0.159
		[0.011,0.308]		[0.201,0.651]		[-0.099,0.418]
**Age**		0.006*		0.006		0.010
		[0.000,0.013]		[-0.003,0.016]		[-0.001,0.021]
**Flood experience**		0.026		0.044		-0.042
		[-0.169,0.221]		[-0.251,0.340]		[-0.382,0.297]
**Migration background**		-0.041		0.139		-0.083
		[-0.240,0.157]		[-0.161,0.439]		[-0.428,0.263]
**Constant**	6.425***	6.189***	5.366***	4.843***	2.776***	2.279***
	[6.327,6.524]	[5.913,6.464]	[5.216,5.515]	[4.426,5.261]	[2.612,2.940]	[1.799,2.759]
**N**	557	468	557	468	557	468

*Notes: This table shows the estimation results of an OLS regression estimating the impact of the treatment PERSPECTIVE-TAKING on participants' perception of the flood victim's situation as an emergency (Perceived need), their appreciation of the person's well-being (Valuing other) and their perceived oneness with the flood victim, measured with the IOS scale (Aaron et al. 1992). 95% confidence intervals are shown in brackets. The symbols *, **, *** indicate significance at p<.05, p<.01, and p<.001, respectively. The coefficient plots at the bottom of Figure 1 are based on model (8), (10) and (12).*

Table A5. Distance Specific Effects of Perspective-Taking on *Behavioral Measures*.

	(1) Donation CLOSE	(2) Donation FAR	(3) Donation pooled	(4) Petition CLOSE	(5) Petition FAR	(6) Petition pooled	(7) Policy approval CLOSE	(8) Policy approval FAR	(9) Policy approval pooled
**Perspective-taking (PT)**	-0.193	0.259	-0.207	-0.168	0.0391	-0.123	0.065	0.029	0.060
	[-0.752,0.366]	[-0.307,0.825]	[-0.757,0.343]	[-0.747,0.411]	[-0.674,0.752]	[-0.695,0.449]	[-0.134,0.263]	[-0.191,0.248]	[-0.145,0.265]
**FAR**			-0.247			-0.844*			-0.093
			[-0.815,0.321]			[-1.498,-0.190]			[-0.304,0.117]
**Perspective-tak.*FAR**			0.455			0.185			-0.021
			[-0.327,1.238]			[-0.722,1.092]			[-0.312,0.270]
**Dispos. income (in EUR)**	-0.00009	0.00003	-0.00001	-0.0004*	-0.0004*	-0.0004**	-0.0001*	-0.0001*	-0.0001**
	[-0.000,0.000]	[-0.000,0.000]	[-0.000,0.000]	[-0.001,-0.000]	[-0.001,-0.000]	[-0.001,-0.000]	[-0.000,-0.000]	[-0.000,-0.000]	[-0.000,-0.000]
**University degree**	0.593*	0.273	0.415*	-0.181	0.220	-0.004	0.058	-0.050	0.010
	[0.014,1.172]	[-0.300,0.847]	[0.012,0.817]	[-0.786,0.424]	[-0.503,0.942]	[-0.464,0.456]	[-0.148,0.264]	[-0.273,0.174]	[-0.141,0.160]
**Gender (not male)**	-0.278	0.242	-0.019	0.381	0.315	0.339	0.297**	0.232*	0.266***
	[-0.864,0.309]	[-0.331,0.815]	[-0.423,0.384]	[-0.210,0.972]	[-0.404,1.033]	[-0.112,0.790]	[0.091,0.503]	[0.008,0.456]	[0.115,0.416]
**Age**	-0.010	-0.004	-0.007	0.016	0.025	0.0190*	0.0003	0.003	0.002
	[-0.033,0.014]	[-0.029,0.021]	[-0.024,0.010]	[-0.007,0.039]	[-0.005,0.055]	[0.001,0.037]	[-0.008,0.008]	[-0.007,0.013]	[-0.005,0.008]
**Flood experience**	0.221	-0.793	-0.268	-0.022	-0.091	-0.035	0.028	-0.149	-0.066
	[-0.558,1.001]	[-1.594,0.008]	[-0.813,0.278]	[-0.849,0.805]	[-1.007,0.825]	[-0.645,0.574]	[-0.256,0.313]	[-0.429,0.132]	[-0.264,0.131]
**Migration background**	-0.220	0.207	-0.020	-0.396	0.523	0.030	-0.111	0.075	-0.020
	[-1.041,0.601]	[-0.527,0.941]	[-0.557,0.518]	[-1.248,0.455]	[-0.351,1.396]	[-0.579,0.639]	[-0.395,0.173]	[-0.214,0.364]	[-0.220,0.180]
**Constant**	-0.279	-0.923	-0.479	-0.730	-2.192**	-0.987*	0.564**	0.443*	0.548***
	[-1.357,0.800]	[-1.988,0.142]	[-1.280,0.321]	[-1.813,0.352]	[-3.550,-0.834]	[-1.861,-0.112]	[0.182,0.945]	[0.030,0.855]	[0.250,0.846]
**N**	236	232	468	236	232	468	236	232	468
**Equality test** for PT btw. CLOSE and FAR	$\chi2(1) = 1.24, p = 0.266$			$\chi2(1) = 0.19, p = 0.663$			$\chi2(1) = 0.06, p = 0.810$		

*Notes: This table shows the estimation results from regressing the impact of the treatment PERSPECTIVE-TAKING (PT) on the willingness to give up own resource (Donation), provide an email address to sign a petition (Petition) and to support structural change (Policy approval). Models (1)–(6) show the coefficients of logit regressions on the likelihood of making a donation or providing the email address. Models (7)-(9) are based on an ordinary least squares regression model estimating the effect of PT on the average approval of 12 realistic policy measures for climate protection in Germany. 95% confidence intervals are shown in brackets. The symbols *, **, *** indicate significance at p<.05, p<.01, and p<.001, respectively. The coefficient plots at the top of Figure 2 are based on model (1)+(2), (4)+(5) and (7)+(8). Models (3), (6) and (9) present the pooled regressions with interaction terms as alternative models for assessing the impact of distance. The results are robust to both specifications.*

Table A6. Additional estimations of the Effect of Perspective-Taking on Donation behavior measured as *continuous* variable

	(1) Donation amount	(2) Donation amount	(3) Donation amount CLOSE	(4) Donation amount FAR	(5) Donation amount pooled
**Perspective-taking (PT)**	-0.008	-0.020	-0.171	0.135	-0.173
	[-0.279,0.263]	[-0.315,0.276]	[-0.596,0.254]	[-0.285,0.554]	[-0.589,0.243]
**FAR**					-0.173
					[-0.601,0.254]
**Perspective-tak.*FAR**					0.311
					[-0.279,0.901]
**Dispos. income (in EUR)**		-0.00006	-0.0001	0.00003	-0.00006
		[-0.000,0.000]	[-0.000,0.000]	[-0.000,0.000]	[-0.000,0.000]
**University degree**		0.352*	0.531*	0.177	0.348*
		[0.048,0.656]	[0.088,0.973]	[-0.249,0.604]	[0.043,0.653
**Gender (not male)**		0.021	-0.113	0.154	0.030
		[-0.282,0.325]	[-0.555,0.329]	[-0.273,0.582]	[-0.275,0.334]
**Age**		-0.002	-0.002	-0.005	-0.003
		[-0.015,0.010]	[-0.019,0.016]	[-0.023,0.014]	[-0.016,0.010]
**Flood experience**		-0.241	-0.048	-0.466	-0.243
		[-0.640,0.157]	[-0.657,0.562]	[-1.001,0.070]	[-0.643,0.157]
**Migration background**		-0.043	-0.362	0.228	-0.053
		[-0.448,0.362]	[-0.971,0.248]	[-0.324,0.779]	[-0.459,0.353]
**Constant**	0.966***	1.029***	1.259**	0.884*	1.128***
	[0.771,1.162]	[0.466,1.592]	[0.441,2.078]	[0.096,1.671]	[0.524,1.731]
**N**	557	468	236	232	468
**Equality test** for PT btw. CLOSE and FAR			$\chi2(1) = 1.04$, p = 0.308		

*Notes: This table shows the OLS regression result from estimating the impact of the treatment PERSPECTIVE-TAKING on participants' willingness to give up own resources measured as donation amounts. 95% confidence intervals are shown in brackets. The symbols *, **, *** indicate significance at p<.05, p<.01, and p<.001, respectively. Model (5) presents the pooled regressions with interaction terms as alternative models for assessing the impact of distance*

Table A7. Distance-Specific Effect of Perspective-Taking on *Mediator Variables.*

	**(10) Perceived need** CLOSE	**(11) Perceived need** FAR	**(12) Perceived need** pooled	**(13) Valuing other** CLOSE	**(14) Valuing other** FAR
**Perspective-taking (PT)**	0.074	0.139	0.084	0.630***	0.395*
	[-0.115,0.263]	[-0.085,0.363]	[-0.120,0.287]	[0.344,0.916]	[0.056,0.734]
**FAR**			0.006		
			[-0.203,0.216]		
**Perspective-tak.*FAR**			0.0610		
			[-0.228,0.350]		
**Dispos. income (in EUR)**	0.00001	-0.00003	-0.000003	0.00005	0.00005
	[-0.000,0.000]	[-0.000,0.000]	[-0.000,0.000]	[-0.000,0.000]	[-0.000,0.000]
**University degree**	0.028	-0.093	-0.0270	-0.105	-0.411*
	[-0.168,0.225]	[-0.321,0.135]	[-0.176,0.123]	[-0.404,0.193]	[-0.756,-0.066]
**Gender (not male)**	0.163	0.147	0.160*	0.349*	0.478**
	[-0.033,0.359]	[-0.082,0.375]	[0.011,0.309]	[0.052,0.646]	[0.132,0.823]
**Age**	0.002	0.012*	0.006*	0.002	0.011
	[-0.006,0.010]	[0.002,0.022]	[0.000,0.013]	[-0.009,0.014]	[-0.004,0.026]
**Flood experience**	0.034	-0.003	0.022	0.136	-0.033
	[-0.236,0.305]	[-0.289,0.283]	[-0.174,0.218]	[-0.274,0.546]	[-0.466,0.399]
**Migration background**	-0.233	0.139	-0.046	0.002	0.323
	[-0.503,0.038]	[-0.156,0.434]	[-0.245,0.153]	[-0.408,0.412]	[-0.123,0.769]
**Constant**	6.323***	6.069***	6.191***	4.952***	4.702***
	[5.960,6.686]	[5.648,6.490]	[5.896,6.487]	[4.401,5.503]	[4.066,5.339]
**N**	236	232	468	236	232
**Equality test** for PT btw. CLOSE and FAR	$\chi2(1) = 0.19$, p = 0.666			$\chi2(1) = 1.13$, p = 0.288	

*Notes: This table shows the estimation results of an OLS regression estimating the impact of the treatment PERSPECTIVE-TAKING on participants' perception of the flood victim's situation as an emergency (Perceived need - Model (7)-(8)), their appreciation of the person's well-being (Valuing other - Model (9)-(10)) and their perceived oneness with the flood victim (Oneness - Model (11)-(12)), measured with the IOS scale (Aaron et al. 1992). 95% confidence intervals are shown in brackets. The symbols *, **, *** indicate significance at p<.05, p<.01, and p<.001, respectively. The coefficient plots at the bottom of Figure 2 are based on these models. Model (12), (15) and (18) present the pooled regressions with interaction terms as alternative models for assessing the impact of distance.*

Table A7. Distance-Specific Effect of Perspective-Taking on *Mediator Variables* ***(cont.)***

	(15) Valuing other	(16) Oneness	(17) Oneness	(18) Oneness
	pooled	CLOSE	FAR	pooled
**Perspective-taking (PT)**	0.630***	0.591***	0.119	0.601***
	[0.323,0.938]	[0.253,0.928]	[-0.247,0.485]	[0.253,0.948]
**FAR**	-0.00484			-0.251
	[-0.321,0.312]			[-0.609,0.106]
**Perspective-tak.*FAR**	-0.234			-0.472
	[-0.671,0.203]			[-0.966,0.021]
**Dispos. income (in EUR)**	0.00006	0.0001	0.0001	0.0001
	[-0.000,0.000]	[-0.000,0.000]	[-0.000,0.000]	[-0.000,0.000]
**University degree**	-0.260*	-0.089	-0.304	-0.191
	[-0.486,-0.035]	[-0.440,0.263]	[-0.677,0.068]	[-0.446,0.064]
**Gender (not male)**	0.424***	0.096	0.226	0.161
	[0.198,0.649]	[-0.255,0.446]	[-0.148,0.599]	[-0.093,0.416]
**Age**	0.006	0.002	0.019*	0.010
	[-0.003,0.016]	[-0.012,0.016]	[0.002,0.035]	[-0.001,0.020]
**Flood experience**	0.057	0.242	-0.237	0.003
	[-0.239,0.353]	[-0.241,0.726]	[-0.704,0.231]	[-0.331,0.337]
**Migration background**	0.154	-0.415	0.341	-0.039
	[-0.147,0.455]	[-0.899,0.068]	[-0.141,0.823]	[-0.378,0.301]
**Constant**	4.825***	2.622***	1.822***	2.343***
	[4.379,5.272]	[1.973,3.272]	[1.134,2.510]	[1.838,2.848]
**N**	468	236	232	468
**Equality test** for PT btw. CLOSE and FAR	$\chi2(1) = 3.60$, p = 0.058			

Table A8. Estimations of the Effect of IRI Subscales Empathic Concern and Perspective-Taking on the Three Behavioral Measures.

	(1) Donation	(2) Petition	(3) Policy approval	(4) Donation	(5) Petition	(6) Policy approval
**IRI subscale Empathic concern**	0.108*	0.158**	0.0685***			
	[0.025,0.191]	[0.062,0.253]	[0.039,0.098]			
**IRI subscale Perspective-tak.**				0.0617	0.0932*	0.0651***
				[-0.017,0.140]	[0.002,0.185]	[0.037,0.094]
**Disposable income (in EUR)**	-0.0000182	-0.000399***	-0.000124***	-0.0000212	-0.000393***	-0.000129***
	[-0.000,0.000]	[-0.001,-0.000]	[-0.000,-0.000]	[-0.000,0.000]	[-0.001,-0.000]	[-0.000,-0.000]
**University degree**	0.444*	0.00564	0.0157	0.419*	-0.0302	0.00288
	[0.038,0.849]	[-0.452,0.463	[-0.131,0.163]	[0.017,0.821]	[-0.483,0.423]	[-0.144,0.150]
**Gender (not male)**	-0.182	0.107	0.166*	-0.0464	0.297	0.244**
	-0.603,0.238]	[-0.357,0.570]	[0.014,0.317]	[-0.448,0.356]	[-0.146,0.740]	[0.098,0.390]
**Age**	-0.00683	0.0204*	0.00109	-0.00636	0.0198*	0.00126
	[-0.024,0.010]	[0.002,0.039]	[-0.005,0.007]	[-0.023,0.011]	[0.002,0.038]	[-0.005,0.007]
**Flood experience**	-0.244	-0.0614	-0.0669	-0.253	-0.0727	-0.0695
	[-0.789,0.302]	[-0.669,0.546]	[-0.259,0.125]	[-0.796,0.291]	[-0.674,0.529]	[-0.262,0.123]
**Migration background**	-0.111	-0.141	-0.0967	-0.0173	-0.0203	-0.0419
	[-0.656,0.434]	[-0.752,0.471]	[-0.295,0.101]	[-0.554,0.520]	[-0.623,0.583]	[-0.238,0.154]
**Constant**	-2.055**	-3.532***	-0.369	-1.482*	-2.690***	-0.376
	[-3.389,-0.722]	[-5.082,-1.982]	[-0.839,0.101]	[-2.807,-0.157]	[-4.212,-1.167]	[-0.853,0.100]
**N**	468	468	468	468	468	468

*Notes: This table shows the estimation results from regressing the impact of the personality traits Empathic Concern and Perspective-taking (IRI subscales) on participants' willingness to give up own resources (Donation), give their email address to sign a petition (Petition) and to support structural change (Policy approval). Models (1), (2), (4) and (5) show the coefficients of logit regressions on the likelihood of making a donation or giving their email address to sign a petition. Models (3) and (6) are based on an ordinary least squares regression model that estimates the effect of Empathic Concern and Perspective-taking on the average approval of 12 realistic policy measures for climate protection in Germany. 95% confidence intervals are shown in brackets. The symbols *, **, *** indicate significance at p<.05, p<.01, and p<.001, respectively.*
